# Polymer Biointerfaces

**DOI:** 10.3390/polym12040793

**Published:** 2020-04-02

**Authors:** Marián Lehocký, Petr Humpolíček

**Affiliations:** 1 Centre of Polymer Systems, University Institute, Tomas Bata University in Zlín, Nam. T.G.M. 5555, 76001 Zlín, Czech Republic; humpolicek@utb.cz; 2 Faculty of Technology, Tomas Bata University in Zlín, Vavreckova 275, 76001 Zlín, Czech Republic

Polymer biointerfaces are considered suitable materials for the improvement and development of numerous applications. The optimization of polymers’ surface properties can control several biological processes, such as cell adhesion, proliferation, viability, and enhanced extracellular matrix secretion at biointerfaces [1,2,3,4,5,6,7,8].

Various routes of polymer biointerfaces preparation are targeted for numerous applications in the biomedical, biochemical, biophysical, biotechnological, food, pharmaceutical, and cosmetic fields [9,10,11,12,13,14,15,16,17,18].

This Special Issue, which consists of 18 articles written by research experts, reports on the most recent research on polymer biointerfaces. Several novel advanced methods related to polymer biointerfaces preparation, modification, analysis, and characterization are introduced.

Firstly, Ozaltin and co-workers treated a polyethylene terephthalate (PET) surface, for use in blood-contacting devices, by DC air plasma and immobilized fucoidan from *Fucus vesiculosus* (FU) on it, at different pH values in the range of 3–7. FU immobilization onto the PET surface after plasma treatment was found to be optimal at pH 5, as supported by FTIR, SEM, and XPS results, and provided the highest anticoagulant activity, more than 100 s, which indicates that the resulting FU-immobilized sample is an efficient anticoagulant, suitable for blood-contacting PET devices. Surface characterization was carried out by a wettability test, scanning electron microscopy, X-ray photoelectron spectroscopy, and Fourier-transform infrared spectroscopy. The anticoagulation activity of the samples was determined on the basis of prothrombin time, activated partial thromboplastin time, and thrombin time [19].

Smilek et al. prepared a hydrogel from oppositely charged biopolymer polyelectrolyte and surfactant in micellar form, at a surfactant/biopolymer charge ratio at least equal to one. Hyaluronan acted as a negatively charged biopolymer, whereas DEAED (amino-modified dextran) was used as a positively charged biopolymer. The former interacted with Septonex (carbethopendecinium bromide), whereas the latter interacted with sodium dodecylsulphate. The rheological properties of hyaluronan-based hydrogels were mainly dependent on the polymer molecular weight. Surfactant concentration (more precisely, the concentration of micelles at a surfactant/biopolymer charge ratio above 1) showed only a small effect. Surfactant concentration was found to have a much greater effect for DEAED-based hydrogels. The authors focused in more detail on the effects of various processing parameters on the properties of similar gels prepared using a slightly different cationic surfactant (approved for use in pharmaceutical formulations) and also investigated a reversely charged system—a positively charged polyelectrolyte (cationized dextran) and an anionic surfactant [20].

Mokrejš and co-workers introduced the preparation of gelatines from by-product collagen raw materials derived from the slaughter of chicken (chicken feet). Gelatine is a water-soluble protein that is obtained from collagenous raw materials by partial hydrolysis. The technological innovation consists in the biotechnological processing of (purified) feedstock by a commercial food endoprotease, which, in contrast to acidic (type A gelatines) or alkaline (type B gelatines) processing, has a variety of economic, technological, and environmental advantages [21].

Belka et al. contributed with current investigations employing MG63 cells grown on Fe-MEPE (metallo-supramolecular coordination polyelectrolyte) modified substrates, which suggest initiation of osteogenic differentiation by both high cell activity and altered morphology of the cells and/or cluster formation. The obtained results led to the conclusion that these surfaces individually support the specification of cell differentiation toward lineages that correspond to the natural commitment of the particular cell types. The authors, therefore, propose that Fe-MEPEs may be used as a scaffold for the treatment of defects in muscular or bone tissues [22].

Karakurt and collaborators investigated the antibacterial activity and cytotoxicity of immobilized glucosamine (GlcN)/chondroitin sulfate (ChS) on polylactic acid. The antibacterial surface modification of polylactic acid films was achieved through the immobilization of GlcN and ChS on film surfaces via plasma treatment, followed by acrylic acid grafting. It was found that the developed GlcN/ChS-coated polylactic acid films are excellent bactericide agents against representative Gram-positive and Gram-negative bacteria. Plasma-treated films immobilized with ChS and GlcN, separately and in combination, demonstrated bactericidal effect against strains of both bacterial types, and the results also revealed the absence of synergistic effect on antibacterial action for the combination. [23].

Lee and co-workers successfully coated Ti substrates with polycaffeic acid and metallic silver using a facile UV light-assisted method for implant applications. They confirmed that the coating process was successful by SEM and AFM analyses. At the same time, they verified the deposition of polycaffeic acid and metallic silver by confirming the elemental composition through XPS, EDS, and mapping methods, and the physical properties (hydrophilicity) of the samples were verified using water contact angle measurements. In vitro biocompatibility and antibacterial studies showed that polycaffeic acid with metallic silver can inhibit bacterial growth, while proliferation of MC-3T3 cells was observed. Therefore, the obtained results suggest that the introduced approach can be considered as a potential method for functional implant coating in the orthopedic field [24].

Bernal-Ballen et al. examined the development of a bioartificial polymeric material made of polyvinyl alcohol (PVA), chitosan (CHI), and fucoidan (FUC) and the incorporation of ampicillin as an antibacterial agent. The prepared films were tested, and it was elucidated that the bioartificial polymeric material has potential for inducing cell regeneration in vitro. The characterization techniques used in the manuscript indicated that PVA brings water resistibility to the system, whereas CHI and FUC are responsible for creating a porous microstructure, which allows the cells to adhere to and grow within the matrix. The obtained information indicated that PVA, CHI, and FUC are compatible, as evidenced by FTIR spectra and SEM images. The new material is an outstanding candidate for cell regeneration, as a result of the synergic effect that each component provides to the blend. [25].

Vitkova et al. obtained nanofibers containing hyaluronic acid (HA) by solution electrospinning. Two approaches were chosen: co-electrospinning of aqueous blend solutions of hyaluronic acid/polyvinyl alcohol and hyaluronic acid/polyethylene oxide and use of an intermediate solvent for electrospinning of pure hyaluronic acid solutions. The choice of materials was done with regard to their potential utilization for cell cultivation. The influences of polymer concentration, average molecular weight (*M*_w_), viscosity, and solution surface tension were analyzed. HA and PVA were fluorescently labeled in order to examine the electrospun structures using fluorescence confocal microscopy [26].

Dvořáková and co-workers demonstrated a new hydrophilization technique based on plasma deposition of a thin film using mixtures of propane/butane with nitrogen at atmospheric pressure. Unlike a simple plasma treatment, the observed high-surface free energy values were due to the properties of the deposited plasma–polymer nanolayer. Therefore, the wettability improvement did not depend on the substrate material, and the aging of the surface modification was highly reduced. The thin layers of the prepared plasma–polymer exhibit highly stable wetting properties, are smooth, homogeneous, and flexible, and adhere well to the surface of polypropylene substrates. Moreover, they are constituted of essential elements only (C, H, N, O). This makes the presented modified plasma–polymer surfaces interesting for further studies in biological and/or technical applications [27].

Habib et al. grafted ascorbic acid onto a polyethylene surface via plasma treatment in order to improve its antimicrobial effects. Plasma treatment was effectively used as a radical initiator with subsequent incorporation of ascorbic acid, which served as an antimicrobial agent, on the polyethylene surface. This modification was confirmed by the enhanced wettability and adhesion properties. The results showed changes in the wettability, adhesion, and roughness of the polyethylene surface after plasma treatment as well as after ascorbic acid grafting. This is a positive indication of the possibility of grafting ascorbic acid onto polymeric materials using plasma pretreatment, enhancing its antibacterial activity [28].

Skopalová and co-workers studied polyaniline films modified by substances with anticipated anticoagulant activity, sodium dodecylbenzenesulfonate, 2-aminoethane-1-sulfonic acid, and *N*-(2-acetamido)-2-aminoethanesulfonic acid. The hemocompatibility tests conducted on these polyaniline films confirmed the absence of anticoagulation activity, though the functional groups typical of anticoagulation substances were present. Hemocompatibility is an essential prerequisite for the application of materials in the field of biomedicine and biosensing. In addition, mixed ionic and electronic conductivity of conducting polymers is an advantageous property for these applications. The results showed that the anticoagulation activity was highly affected by the presence of suitable functional groups originating from the used heparin-like substances and by the properties of the polyaniline polymer itself [29].

Urbánková et al. used sodium caseinate in order to stabilize emulsions containing bioactive tamanu and black cumin oils. The emulsions were prepared by ultrasound treatment or high-shear homogenization with Ultra-Turrax. The analysis of the oils’ fatty acid composition revealed a higher degree of unsaturation for cumin oil, with higher content of linoleic acid C18:2, which corresponded to the higher iodine value determined for this oil. The antibacterial activities of both oils and of their emulsions were investigated with respect to the growth suppression of common spoilage bacteria, using the disk diffusion method. The oils and selected emulsions were proven to act against Gram-positive strains, mainly against *Staphylococcus aureus* and *Bacillus cereus*. Regrettably, Gram-negative species were fully resistant to their action [30].

Sťahel and co-workers deposited oxazoline-based thin films on glass substrates using atmospheric pressure dielectric barrier discharge. This study presents a new way to produce plasma-polymerized oxazoline polymers, which are a new promising class of polymers for biomedical applications, with antibiofouling properties and good biocompatibility. The authors describe the film preparation procedure. Nitrogen was used as the working gas for the discharge, 2-methyl-2-oxazoline vapors, used as the monomer, were admixed to the nitrogen flow. This gas composition made it possible to obtain a homogeneous discharge, which led to the deposition of homogeneous thin films. To improve the film properties, it was necessary to increase the substrate temperature during the deposition. All deposited films are cytocompatible and exhibited excellent antibacterial properties against *S. epidermidis*, *S. aureus*, and *Escherichia coli* [31].

Shah et al. developed a novel dual crosslinked film for promising future applications in ophthalmology, skin tissue engineering, and wound dressing. Firstly, a collagen/chitosan film was prepared by the solvent casting technique, utilizing two crosslinking agents together, i.e., tannic acid and genipin. The obtained final dual crosslinked film was translucent, thin, and greenish-blue in color. Enzymatic degradation of the films was performed with lysozyme and lipase. Cell adhesion and proliferation were tested using mouse embryonic cell lines, by culturing the cells on the dual crosslinked film. These dual crosslinked polymeric film find their application in ophthalmology, especially as implants for temporary injured cornea and skin tissue regeneration [32].

Vesel and co-workers presented results regarding systematic 2D mapping of surface wettability, which provide an insight in processes responsible for surface activation of the polymer polyethylene terephthalate using a simple atmospheric-pressure plasma jet. The discharge tube was only flushed with Ar gas to get rid of permanent gases, such as nitrogen, oxygen and CO_2_, but the water remained on the surfaces, due to the humidity and laboratory atmosphere. In the case of a rapid activation, a very sharp interphase between the activated and the unaffected surface was observed and explained by the peculiarities of high-impedance discharges sustained in Ar with the presence of impurities from water vapor. The results obtained by X-ray photoelectron spectroscopy confirmed that the activation was a consequence of functionalization with oxygen functional groups [33].

Narimisa et al. applied a plasma source to deposit thin layers under atmospheric pressure. Using different techniques, the effect of process parameters such as applied power, carrier gas flow rate, distance from capillary to the substrate, and treatment time on the deposition efficiency was studied. A high level of monomer fragmentation observed with optical emission spectroscopy, together with the non-uniform distribution of the monomer observed by computational fluid dynamic simulations, was shown to be a reliable indicator of coating quality. By following this research strategy, crucial information regarding the effectiveness of atmospheric-pressure microwave plasma jet for thin film deposition can be revealed [34].

Villamil Ballesteros and co-workers decellularized bovine amniotic membranes using four different protocols, and the differences in terms of decellularization were considered as negligible. All membranes provided DNA concentrations <50 ng/mg, indicating that traces of the nucleic acid were present in the prepared material, although the obtained values were negligible, which implies that the decellularized membranes did not contain native cells from the bovine amniotic membranes. The in vitro biocompatibility of the studied samples was demonstrated. Hence, these matrices may be deemed as a potential scaffold for epithelial tissue regeneration [35].

Finally, Klučáková studied the influence of humic acids on the transport of metal ions and dyes in agarose hydrogels. It was confirmed that humic acids retarded the transport of diffusion probes. Humic acids’ enrichment caused decreases in the values of effective diffusion coefficients, due to their complexation with the diffusion probes. The effect of complexation was selective for a particular diffusion probe. The aim of this study was to investigate the influence of the interactions between humic acids and probes on hydrogels’ release ability, as hydrogels play an important role in the monitoring of the mobility of pollutants in nature as well as in their removal and in water treatment. They are usually based on materials able to absorb water and different pollutants in their structure [36].
